# Temporal changes in *Plasmodium falciparum* reticulocyte binding protein homolog 2b (*PfRh2b*) in Senegal and The Gambia

**DOI:** 10.1186/s12936-019-2868-x

**Published:** 2019-07-16

**Authors:** Cyrille K. Diédhiou, Rahama A. Moussa, Amy K. Bei, Rachel Daniels, Nasserdine Papa Mze, Daouda Ndiaye, Ngor Faye, Dyann Wirth, Alfred Amambua-Ngwa, Souleymane Mboup, Ambroise D. Ahouidi

**Affiliations:** 1 Institute for Health Research, Epidemiological Surveillance and Training (IRESSEF), Dakar, Senegal; 2 Laboratory of Bacteriology and Virology, Hospital Aristide Le Dantec, 7325, Dakar, Senegal; 3 Department of Immunology & Infectious Diseases, Harvard TH Chan School of Public Health, 665 Huntington Avenue, Boston, MA USA; 4 Laboratory of Parasitology Mycology, Aristide Le Dantec Hospital, Faculty of Medicine and Pharmacy, Cheikh Anta Diop University, 5005, Dakar, Senegal; 5 Faculty of Sciences and Technologies, University Cheikh Anta Diop, Dakar, PO Box 5005, Dakar, Senegal; 6 Medical Research Council Unit, The Gambia at LSHTM, Fajara, Banjul, The Gambia

**Keywords:** *Plasmodium falciparum*, *PfRh2b*, SNPs, Senegal, Gambia

## Abstract

**Background:**

The *Plasmodium falciparum* reticulocyte binding protein homolog 2b (PfRh2b) is an important *P. falciparum* merozoite ligand that mediates invasion of erythrocytes by interacting with a chymotrypsin-sensitive “receptor Z”. A large deletion polymorphism is found in the c-terminal ectodomain of this protein in many countries around the world, resulting in a truncated, but expressed protein. The varying frequencies by region suggest that there could be region specific immune selection at this locus. Therefore, this study was designed to determine temporal changes in the *PfRh2b* deletion polymorphism in infected individuals from Thiès (Senegal) and Western Gambia (The Gambia). It was also sought to determine the selective pressures acting at this locus and whether prevalence of the deletion in isolates genotyped by a 24-SNP molecular barcode is linked to background genotype or whether there might be independent selection acting at this locus.

**Methods:**

Infected blood samples were sourced from archives of previous studies conducted between 2007 and 2013 at SLAP clinic in Thiès and from 1984 to 2013 in Western Gambia by MRC Unit at LSHTM, The Gambia. A total of 1380 samples were screened for the dimorphic alleles of the *PfRh2b* using semi-nested Polymerase Chain Reaction PCR. Samples from Thiès were previously barcoded.

**Results:**

In Thiès, a consistent trend of decreasing prevalence of the *PfRh2b* deletion over time was observed: from 66.54% in 2007 and to 38.1% in 2013. In contrast, in Western Gambia, the frequency of the deletion fluctuated over time; it increased between 1984 and 2005 from (58.04%) to (69.33%) and decreased to 47.47% in 2007. Between 2007 and 2012, the prevalence of this deletion increased significantly from 47.47 to 83.02% and finally declined significantly to 57.94% in 2013. Association between the presence of this deletion and age was found in Thiès, however, not in Western Gambia. For the majority of isolates, the *PfRh2b* alleles could be tracked with specific 24-SNP barcoded genotype, indicating a lack of independent selection at this locus.

**Conclusion:**

*PfRh2b* deletion was found in the two countries with varying prevalence during the study period. However, these temporal and spatial variations could be an obstacle to the implementation of this protein as a potential vaccine candidate.

**Electronic supplementary material:**

The online version of this article (10.1186/s12936-019-2868-x) contains supplementary material, which is available to authorized users.

## Background

Despite significant efforts and progress directed towards malaria prevention and control, malaria from *Plasmodium falciparum* parasite infection remains a major global health challenge. This is due to multiple factors, including insecticide resistance in anopheline vectors, the emergence and rapid spread of drug-resistant parasite strains and especially the lack of an effective vaccine. Therefore, the development of an effective malaria vaccine remains critical for malaria eradication. However, efforts at developing a malaria vaccine have been hampered by the extensive genetic diversity in malaria parasite populations and allele specific immunity in endemic human populations [1, 2].

Characterization of genetic polymorphisms in key vaccine antigens of *P. falciparum* will enable a better understanding of the molecular evolution of parasite populations that could affect efficacy of future vaccines [3]. *Plasmodium falciparum* Reticulocyte binding protein homologues (PfRhs) are expressed at the apical surface of invasive merozoite [4–7] and are believed to play a role in the recognition of the erythrocyte and in tight junction formation [8]. Towards design of a vaccine, several studies are looking at the immunity and diversity of the PfRh family of proteins [6, 8–13]. There are five functional *PfRh* genes: *P*. *falciparum* Reticulocyte-binding protein homolog 1 (*PfRh1*) [14, 15], *PfRh2a*, *PfRh2b* [16–18], *PfRh4* [19, 20], and *PfRh5* [21–23]. Two members of the *P. falciparum* Rh ligands, PfRh2a and PfRh2b, are important mediators of parasite invasion. PfRh2b mediates invasion by interacting with a chymotrypsin-sensitive erythrocyte receptor Z [24]. These proteins are currently being assessed as invasion-blocking vaccine candidates [11, 25]. However, they differ structurally at the c-terminal.

The c-terminal region of *PfRh2b* gene presents a large structural polymorphism (0.58 kb) and this was found at high frequencies in field isolates from different areas of Africa [10, 26, 27]. The prevalence of this deletion was characterized for the first time in 2006 in parasite populations from different areas [10]. However, the prevalence and evolution of the PfRh2b protein as malaria declines remains unknown. Understanding the changes over time in the frequencies of allelic variants in potential vaccine candidate will be important, as these changes could affect the efficacy of a candidate vaccine. Therefore, it is important to assess temporal changes in the *PfRh2b* gene and prevalence of the dimorphic alleles *PfRh2b* deletion (*PfRh2bdel*) over time in endemic populations.

As shown for Thiès, in Senegal, neutral single nucleotide polymorphism (SNP) markers can sensitively determine temporal changes in *P. falciparum* genotypic diversity [28]. There are extensive hotspots of diversity across the genome, mostly within genes exposed on the surface of the parasite and erythrocyte, including invasion ligands. These ligands are a target of immune responses and remain a major focus for development of a blood stage vaccine [29, 30].

As the frequency of genotypes changes as well as the *PfRh2b* alleles, the prevalence of the deletion was determined using molecular barcode to examine whether allelic frequencies are strictly related to barcode haplotype clusters or independent selection at the *PfRh2b* locus could be driving frequencies in the population. This provides data relevant for further consideration of PfRh2b and other structurally variant proteins as targets for vaccine development.

## Methods

### Study sites and *Plasmodium falciparum* field isolates

Archived *P. falciparum* DNA samples from infected blood samples of consenting individuals were sourced from previous studies conducted between 2007 and 2013 at the Service de Lutte Anti-Parasitaire (SLAP) clinic in Thiès (70 km from Dakar, the capital city of Senegal) and from 1984 to 2013 in Western Gambia. Overall, malaria prevalence is moderate in The Gambia with high seasonal transmission [31, 32]. In contrast, Thiès is characterized by a perennial hypo-endemic transmission. These studies had received ethical approval from the Institutional Review Boards of the Harvard School of Public Health, the Ethics Committee of the Ministry of Health in Senegal and the Joint Gambian Government/MRCG Ethics Committee. A total of 1380 (849 from Thiès and 531 from Western Gambia) *P. falciparum* malaria infected blood samples were analysed. Among the Thiès samples, 580 that has been previously genotyped using a molecular barcode of 24 SNPs by Daniels et al. [33] were used for determining the association between alleles of *PfRh2b* and the specific barcode of parasite.

### *PfRh2b* genotyping

Semi-nested PCR method, as described previously [10], was used to amplify an *PfRh2b* gene fragment including the deletion in all extracted DNA samples. A positive control (*P. falciparum* laboratory cloned line 3D7) and negative control (reagent grade water) were included in all PCR amplifications. The size of the PCR products was estimated using Gene Ruler 100 bp DNA ladder marker (Quick Load^®^, 100pb DNA Ladder).

### Statistical analysis

For continuous variables median and interquartile range were calculated, while for categorical variables, the proportion or prevalence of the outcome with 95% CI was calculated.

For each year, to take into account the prevalence in mixed genotype isolates, the prevalence of *PfRh2b* full-length and *PfRh2b* deletion was calculated as follows:

(number of *PfRh2b allele* isolates + (0.5 * number of mixed isolates))/total number of isolates [10]. The Chi square linear trends was used to determine if the differences in frequency over the years was statistically significant. Differences between groups were assessed using Mann–Whitney U-test. Wright’s fixation index (*Fst*) was also calculated to assess the extent of genetic differentiation of *PfRh2b* polymorphism in Thiès and Western Gambia over time.

## Results

### Evolution of the prevalence of *PfRh2b* deletion in fields isolates from Thiès and Western Gambia

**Fig. 1:**
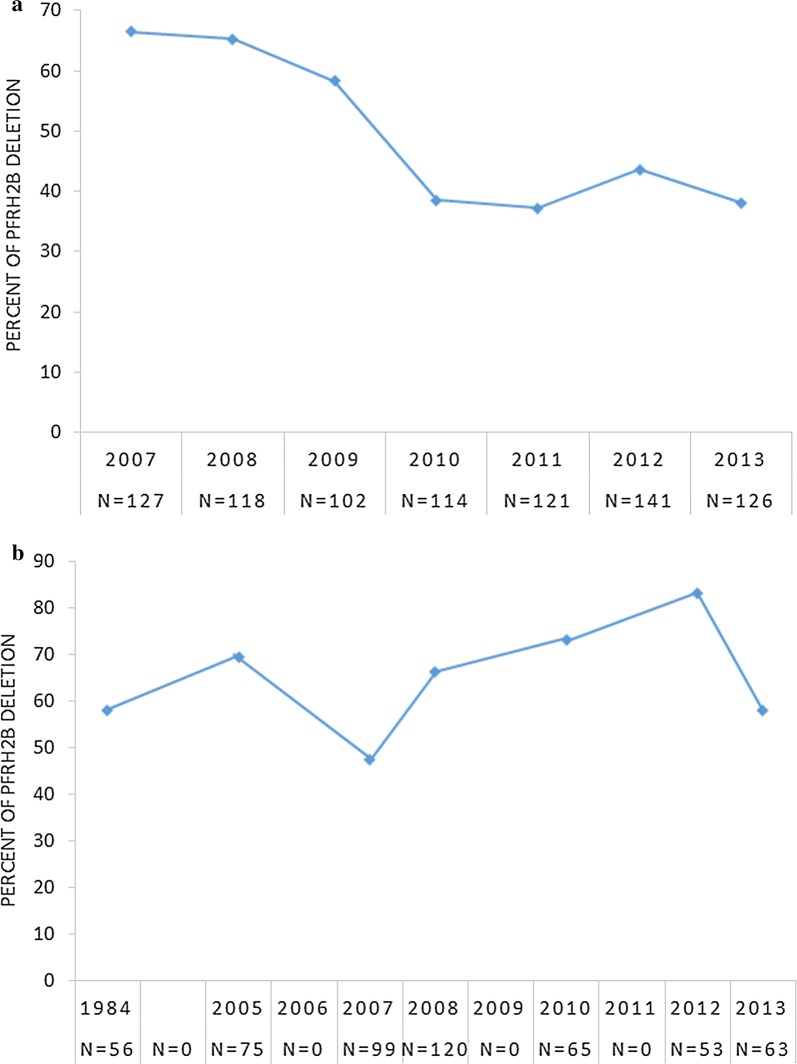
Evolution of *PfRh2b* deletion in Thiès (**a**) and Western Gambia (**b**). Scheme showing the variation in the prevalence of *PfRh2b* deletion over the time, including mixed infections (*PfRh2* full-length and *PfRh2b* deletion) in both areas using formula described previously by Ahouidi et al. [10]. Alleles frequencies of the dimorphic *PfRh2b* gene were determined using semi-nested Polymerase Chain Reaction (PCR). **a** A decrease of *PfRh2b* deletion from 2007 to 2013 in Thiès was noted. **b** A fluctuation of this deletion from 1984 to 2013 in The Gambia was observed. N, number of isolates; Del, deletion

In Thiès, the prevalence of the deletion decreased significantly from 66.54% in 2007 to 38.1% in 2013 (P < 0.0001). This decline was not homogeneous with the presence of a peak in 2012, where *PfRh2bdel* form was present in 43.62% of infections (Fig. 1a).

In Western Gambia, temporal variation in prevalence in *PfRh2b* deletion was observed between 1984 and 2013. The prevalence of *PfRh2b* deletion increased between 1984 and 2005 from (58.04%) to (69.33%) (P = 0.03). From 2005 to 2007 there was a decline of the deletion to (47.47%) (P = 0.004). Between 2007 and 2012, the prevalence of this deletion increased significantly from 47.47 to 83.02% (P = 0.00005) and finally declined significantly to 57.94% in 2013 (P = 0.001) (Fig. 1b). Since mixed infections are uncommon in endemic populations, the frequency of infections with both deleted and full-length parasites (mixed) was determined. The result was highest in 2007 (0.10) and lowest in 2009 (0.02) in Thiès. In Western Gambia, mixed infections were most common in the earliest population from 1984 (0.23) and lowest in 2008 with (0.04) (Additional file 1: Table S1).

Overall, a significant decrease of the prevalence of *PfRh2b* deletion from 2007 to 2013 in Thiès (P < 0.0001) and fluctuating prevalence from 1984 to 2013 in Western Gambia were observed.

### Prevalence of *PfRh2b* deletion according to age in Thiès and western Gambia

The presence of the deletion at high frequency in the general population and the acquisition of antibodies in an age-dependent manner against the c-terminal region of *PfRh2b* [10], have raised the interest to determine the prevalence of the deletion by age-group to see if the deletion would be more frequent in adults since they are long exposed. Thus, among the 1380 *Plasmodium* isolates assayed, 934 samples were available for age data and were analysed (651 from Thiès and 283 from Western Gambia). A median age was used to divide each population into two numerically equal groups.

**Fig. 2:**
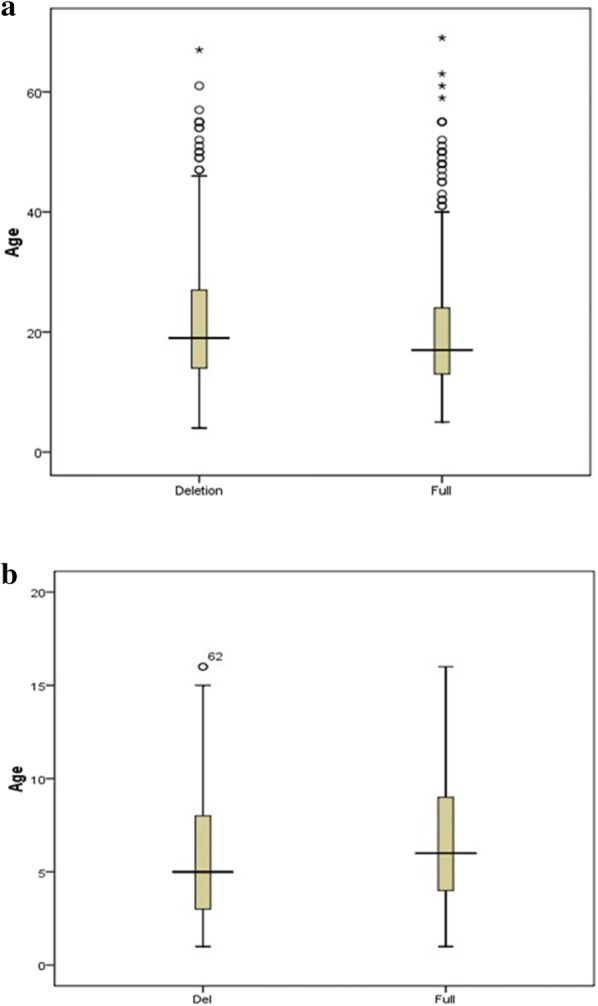
Percentage of *PfRh2b* deletion according to age groups in Thiès (**a**) and Western Gambia (**b**). Variation in the presence of *PfRh2b* deletion according to age of patients from two distinct populations. **a** In Thiès, the median age of the 651 Patients was 18 years. A significant difference in the presence of *PfRh2b* deletion was observed (P = 0,037). **b** In Western Gambia, the median age of the 283 Patients was 5 years. No difference in the presence of *PfRh2b* deletion was found (P = 0.316). P, P value; ns, not significant; Del, deletion

In Thiès, since the study population mainly consists of adults (62%), the median age of the 651 patients was 18 years. Thus, the *PfRh2b* deletion form was less common in children compared to older patients (Fig. 2a). Using the Mann–Whitney U-test, a significant difference in the presence of *PfRh2b* deletion was found (P = 0.037), suggesting that there is an association between age and the presence of this deletion in Thiès.

In contrast, in Western Gambia, the population study is consisting of children (under 16 years of age); the median age of the 283 patients was 5 years. The *PfRh2b* deletion was more common in younger children compared to older children (Fig. 2b), but the difference is not significant by the Mann–Whitney U-test (P = 0.316).

### Temporal differentiation of *PfRh2b* polymorphisms in Senegal and Gambia populations

Genetic diversity of the genes in natural parasites populations is a real obstacle for the validation of potential vaccine candidate. In this study, the degree of divergence to which *PfRh2b* gene is subject to selection was estimated by calculating *Fst* from the allelic frequencies of this locus according to years.

In Senegal the *Fst* value obtained from 2007 to 2013 was high (0.09), suggesting high degree of allelic divergence in this region over the time. In contrast, the *Fst* value obtained in The Gambia from 1984 to 2013 was low (0.057), suggesting less genetic differentiation of these alleles in this area over the time (Table 1).

**Table 1:** Allele frequencies used to calculate *Fst* according to years

Years	THIES			WESTERN GAMBIA		
	*PfRh2bdel*	*PfRh2bfull*	Gene diversity (h)	*PfRh2bdel*	*PfRh2bfull*	Gene diversity (h)
1984	–	–		0.58	0.42	0.487
2005	–	–		0.69	0.31	0.428
2007	0.67	0.33	0.442	0.47	0.53	0.498
2008	0.65	0.35	0.455	0.66	0.34	0.449
2009	0.58	0.42	0.487	–	–	–
2010	0.39	0.61	0.372	0.73	0.27	0.394
2011	0.37	0.63	0.466	–	–	–
2012	0.44	0.56	0.493	0.83	0.17	0.282
2013	0.38	0.62	0.471	0.58	0.42	0.487
Average	0.497	0.503	0.455	0.648	0.351	0.432
	*Fst* = 0.09			*Fst* = 0.057		

Temporal differentiation of *PfRh2b* polymorphism in Thiès and Western Gambia populations. *Fst* value was calculated to assess the extent of temporal variation in the frequencies of *PfRh2b* alleles in both areas. The overall estimated value of *Fst* from 2007 to 2013 in Thiès was 0.09. In Western Gambia the *Fst* value from 1984 to 2013 was 0.057

*Fst*, wright’s fixation index; h, gene diversity

### Prevalence of *PfRh2b* in isolates grouped by molecular barcode

It has been observed by barcoding in Thiès that the parasite, having undergone various interventions and pressures, has adapted by evolving towards to a type of parasite [28]. It is also assumed that parasite, initially having the *PfRh2b* full-length, has adapted by presenting the deletion following the different pressures. To test the hypothesis that the parasite with *PfRh2b* deletion may be linked by specific cluster, the prevalence of *PfRh2b* deletion according to molecular barcode was determined. Among the samples that were analysed for *PfRh2b* polymorphism, 580 from Thiès had been previously genotyped using the 24-SNP barcode [33] and the results were classified into two groups: a group named “cluster” which includes the samples having similarities with at least one other isolate in their nucleotide sequences and a group called “unique” grouping parasites with unique SNP barcode. 41.73% (242/580) of samples belonged to the cluster-group and 58.27% (338/580) were in the unique group (Table 2).

**Table 2:** Nucleotide sequence of haplotype clusters in Thiès

Haplotype cluster	Molecular barcodes	N (%)	N (isolats)	*PfRh2bdel* (%)	*PfRh2bfull* (%)
Haplotype cluster 36	CACTGCAGACCGCACCCAAGCCTG	0.345	2	100	0
Haplotype cluster 38	CACTCGAGATCGTCACCACGCTTG	0.345	2	0	100
Haplotype cluster 45	TATTCCGGTCCGTCCCCTCGCTTG	0.345	2	100	0
Haplotype cluster 51	TACTCCGGTTCGCACACACGACTG	0.345	2	100	0
Haplotype cluster 49	TATTCGAAATCGCACCCTAGATTG	0.345	2	100	0
Haplotype cluster 48	TACTCCAGTCCATACACACGATTG	0.345	2	100	0
Haplotype cluster 46	TACTGCAGATTGTACCCAAAACTG	0.345	2	50	50
Haplotype cluster 57	CACTGCGGATTGTACCTAAGACTG	0.345	2	50	50
Haplotype cluster 54	CGCTCCAGACTACACCCTAAACTG	0.345	2	0	100
Haplotype cluster 53	TACTCCGGATTGTCACCAAGACTG	0.345	2	100	0
Haplotype cluster 59	TACTCCGGTTTATACCTTAGACTG	0.345	2	0	100
Haplotype cluster 61	TACCGGAGTCCGTACCTAAGCCTG	0.345	2	0	100
Haplotype cluster 15	TACTCCGGTTCGTAAACTCGCCTG	0.345	2	50	50
Haplotype cluster 63	TACTCCAGACCGCCCCTAAAATTG	0.345	2	0	100
Haplotype cluster 9	TATTCCAGATXGCAACTTCGACTG	0.345	2	100	0
Haplotype cluster 62	TACTCGAGACTGCNCATACACTTG	0.345	2	0	100
Haplotype cluster 13	TACTCGAAACTXCCCATAAGCTTG	0.345	2	0	100
Haplotype cluster 68	TACCCCGGACCACCAATAAGACTG	0.345	2	0	100
Haplotype cluster 69	TACTGGGATCCGCACCTAAGACTG	0.345	2	0	100
Haplotype cluster 67	CACTCCGGATTGCCACTTAGATTG	0.345	2	50	50
Haplotype cluster 70	TATTCCGGACXACACACTAGCTTG	0.345	2	0	100
Haplotype cluster 22	TACTCCGGATCGCACCCTAGATTG	0.345	2	50	50
Haplotype cluster 74	TACTCCAGACTATCCATTCGATTG	0.345	2	50	50
Haplotype cluster 71	CACTCGGGATTXCCACTAAGCTTG	0.345	2	0	100
Haplotype cluster 80	CATTCCAGTCCXCCAATAAGATTG	0.345	2	0	100
Haplotype cluster 72	TATTGGGGATCGCAACCAAGATTG	0.345	2	100	0
Haplotype cluster 77	TACTGGAGTCCGTACCTTAGCTTG	0.345	2	50	50
Haplotype cluster 97	CACTCGAAATXATACCTTAGCTTG	0.345	2	50	50
Haplotype cluster 87	TACTCGGGTCTATAAATAAGACTG	0.345	2	0	100
Haplotype cluster 89	TACTCGAGTTTATACCTTAGACTG	0.345	2	0	100
Haplotype cluster 92	TATTGCAGTCCXCAAATAAGCTTG	0.345	2	0	100
Haplotype cluster 84	CACTCCAGTCCACCACNTAGATTG	0.345	2	100	0
Haplotype cluster 96	TATTCCAGACCGCACATTAGCCTG	0.345	2	50	50
Haplotype cluster 93	TACTCCAGTCCGTCACTTAGACTG	0.345	2	100	0
Haplotype cluster 44	TACTCCAGACTACAACTACGCCTG	0.345	2	0	100
Haplotype cluster 43	TATTCCAGATTGCAACTTCGCCTG	0.517	3	100	0
Haplotype cluster 58	CACTCGAGTTXACAACCTAGCCTG	0.517	3	33	67
Haplotype cluster 7	CACTCCGGATTGCCACTAAGATTG	0.517	3	33	67
Haplotype cluster 19	TATTCGAGTCTACACCTTCACTTG	0.517	3	100	0
Haplotype cluster 21	TACCCCGGTCCACCACTAAAATTG	0.517	3	0	100
Haplotype cluster 23	CACCCGAGTCCACCAACAAGACTG	0.517	3	0	100
Haplotype cluster 95	CACCCCGAATCXCACCTAAGACTG	0.517	3	0	100
Haplotype cluster 99	TACTCCGAACTGCACATTAGATTG	0.517	3	100	0
Haplotype cluster 55	TACTCCGGTTTGCACACACGACTG	0.69	4	100	0
Haplotype cluster 64	TACTCGAGATXATACATACACTTG	0.69	4	0	100
Haplotype cluster 10	CATTGCGATCTGCAACCTAAACTG	0.69	4	100	0
Haplotype cluster 24	CATTCCAGTCCXCCCATTAGATTG	0.69	4	25	75
Haplotype cluster 81	TACTCCAGATCGCACCCAAGCCTG	0.69	4	75	25
Haplotype cluster 98	CACTCGAGTTTACAACTAAGATTG	0.69	4	25	75
Haplotype cluster 5	TACTCGAAACTGCCCATAAGCTTG	0.69	4	0	100
Haplotype cluster 65	CACTCCAAATCGTACCTTAGATTG	0.862	5	100	0
Haplotype cluster 8	TACCCCGGTCCACACCTTAACTTG	0.862	5	100	0
Haplotype cluster 11	TACTCGAGATCATACATACACTTG	0.862	5	0	100
Haplotype cluster 12	CACTGCGATCTGCAACCTAAACTG	0.862	5	100	0
Haplotype cluster 6	CATTCCAGTCCGCCAATAAGATTG	1.034	6	0	100
Haplotype cluster 26	CACTCCAGTCCGTCACCAAGATTG	1.034	6	17	83
Haplotype cluster 17	TACCCCGGTCCACCAATAAGATTG	1.207	7	0	100
Haplotype cluster 16	TACTCCAGATTACAACCTAGCCTG	1.207	7	100	0
Haplotype cluster 66	TGTTCCAGTTTATCACCACGCCTG	1.379	8	12.50	87.50
Haplotype cluster 18	TATTCCAGTCCACCCATAAGACTG	1.552	9	89	11
Haplotype cluster 4	TACTCCGGTTXGCACACACGACTG	2.586	15	100	0
Haplotype cluster 29	TACCCCGGTCCACCAATAAGACTG	7.241	42	9.50	90.50
	UNIQUES	58.27	338	49.11	50.89

N, number of isolates; *PfRh2bdel*, deletion present; *PfRh2bfull*, full-length sequence

No significant difference in the frequency of *PfRh2b* deletion between the cluster-group and the unique group was found (P = 0.5014). Among the 338 isolates having unique barcodes, 166 (49.11%) had the deletion and 172 (50.89%) had the full-length fragment of *PfRh2b* gene. Therefore, both alleles *PfRh2b* deletion and *PfRh2b* full-length, had a similar distribution within the unique genotype group. Furthermore, all the 242 isolates belonging to the cluster-group were also tested to assess the frequency of *PfRh2b* alleles. In this group, 62 distinct subgroups were identified. The results showed that 20 haplotype clusters had only the deletion variant of *PfRh2b* gene, 24 subgroups had only the full-length fragment and the remaining 18 subgroups were parasites having the deletion and full-length (Table 2).

**Fig. 3:**
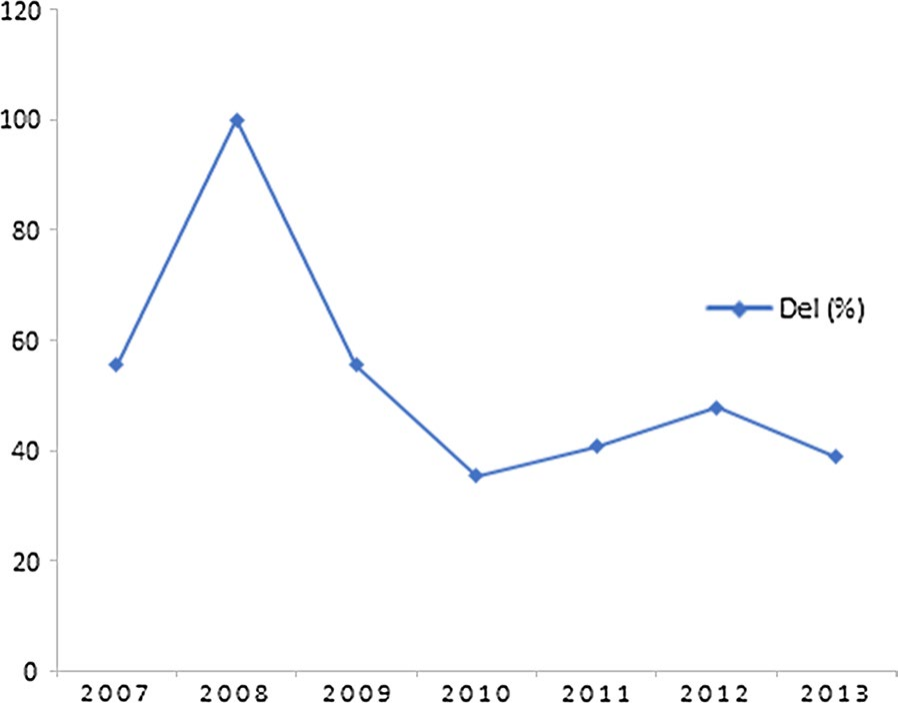
Evolution over time of PfRh2b deletion in haplotype clusters having only the deletion in Thiès

**Fig. 4:**
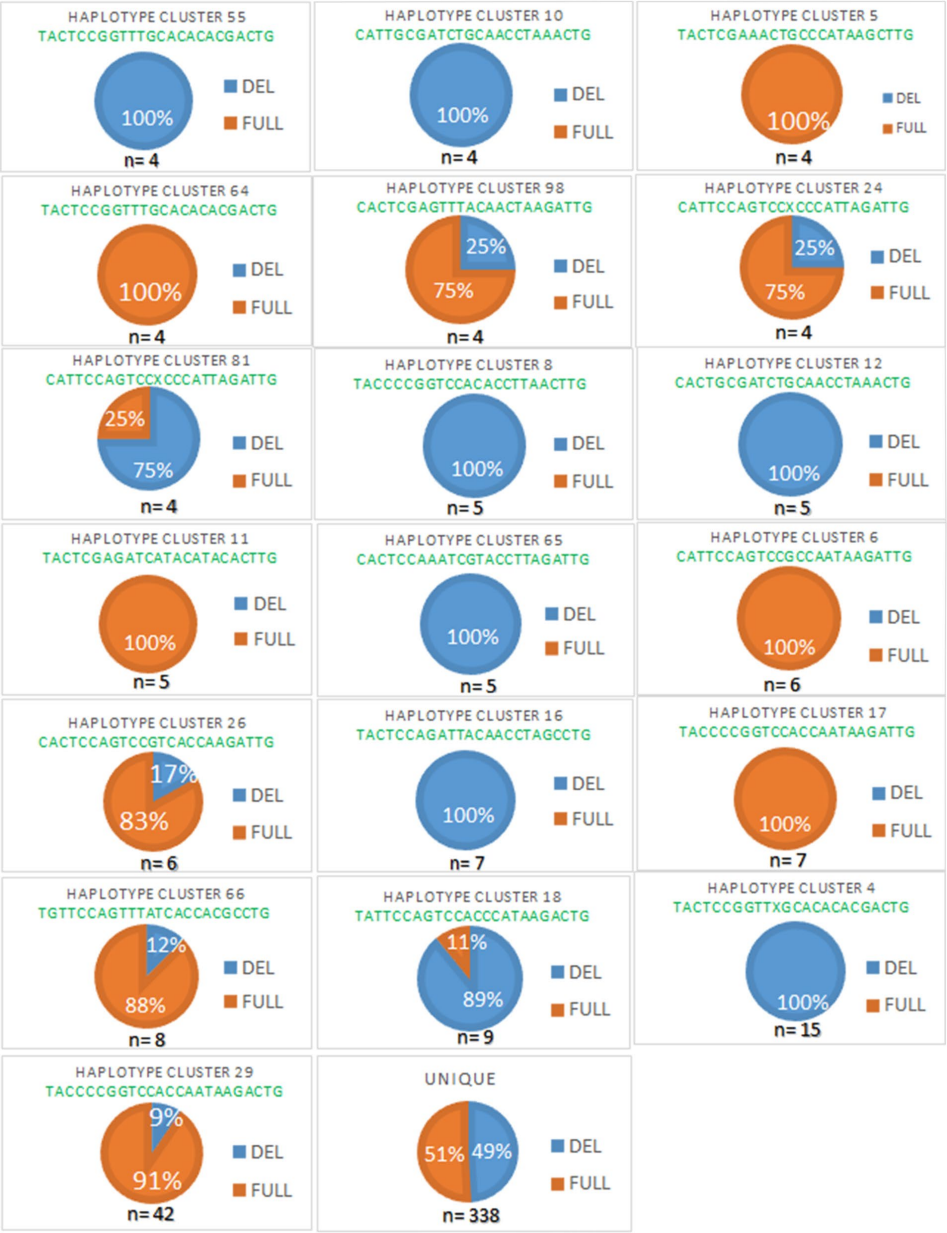
Percentage of isolates of cluster-group and unique based on *PfRh2b* polymorphism in Thiès. Scheme showing the association between the polymorphism of *PfRh2b* gene and specific barcode of parasite. “Haplotype clusters” which had n = 4 samples were represented. Haplotype cluster 55; 10; 65; 8; 12; 16 and 4 had only the deletion of *PfRh2b* gene (Blue pie chart); Haplotype cluster 64; 5; 11; 6 and 17 had only the full-length fragment (orange pie chart). The remaining (Haplotype cluster 24; 31; 98; 26; 66; 18 and 29) were parasites having deletion and full-length alleles. The last pie chart (with n = 338) represented the parasites with unique barcode. Del, deletion; Full, full-length sequence; n, number of isolates

**Fig. 5:**
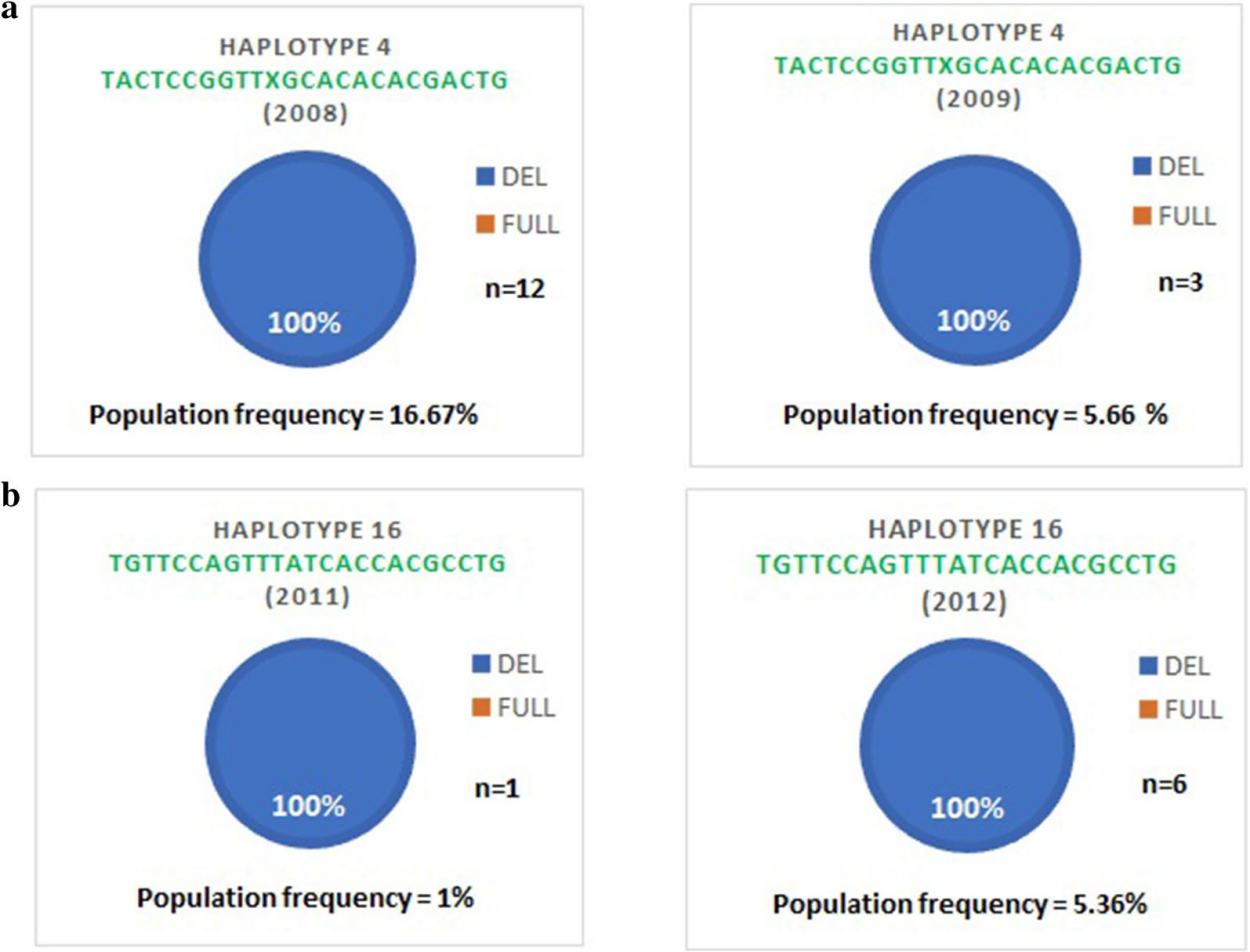
Frequency of deletion by year in haplotype clusters 4 and 16. Monitoring of the population frequency and *PfRh2b* alleles frequencies of haplotype cluster 4 and 16 over the time. **a** Haplotype cluster 4 was found in 2008 (n = 12) and 2009 (n = 3), but not in the other years. **b** Haplotype cluster 16 was present in 2011 (n = 1) and 2012 (n = 6), but not in the other years. Del, deletion; Full, full-length sequence; n, number of isolates

The number of samples is differently distributed in each of the 20 haplotype clusters with the deletion, and this distribution varies also over time (Table 3). Evolution over time of the 20 haplotype clusters containing only the PfRh2b deletion shows a decrease of the prevalence between 2008 and 2010 as well as a slight increase between 2010 and 2013 (Fig. 3). Since the numbers in the majority of the cluster group with the PfRh2b deletion are limited, haplotypes with n > 5 were examined further (haplotype 4 and 16) (Fig. 4). Haplotype cluster 4 with the deletion was found in 2008 (n = 12) and 2009 (n = 3), but not in the other years. Meanwhile, haplotype cluster 16 with the deletion was present in 2011 (n = 1), and 2012 (n = 6), but not in the other years (Fig. 5). The analysis of the results over time suggests that the frequency of *PfRh2b* deletion is related to the presence of some haplotype clusters in this population.

**Table 3:**
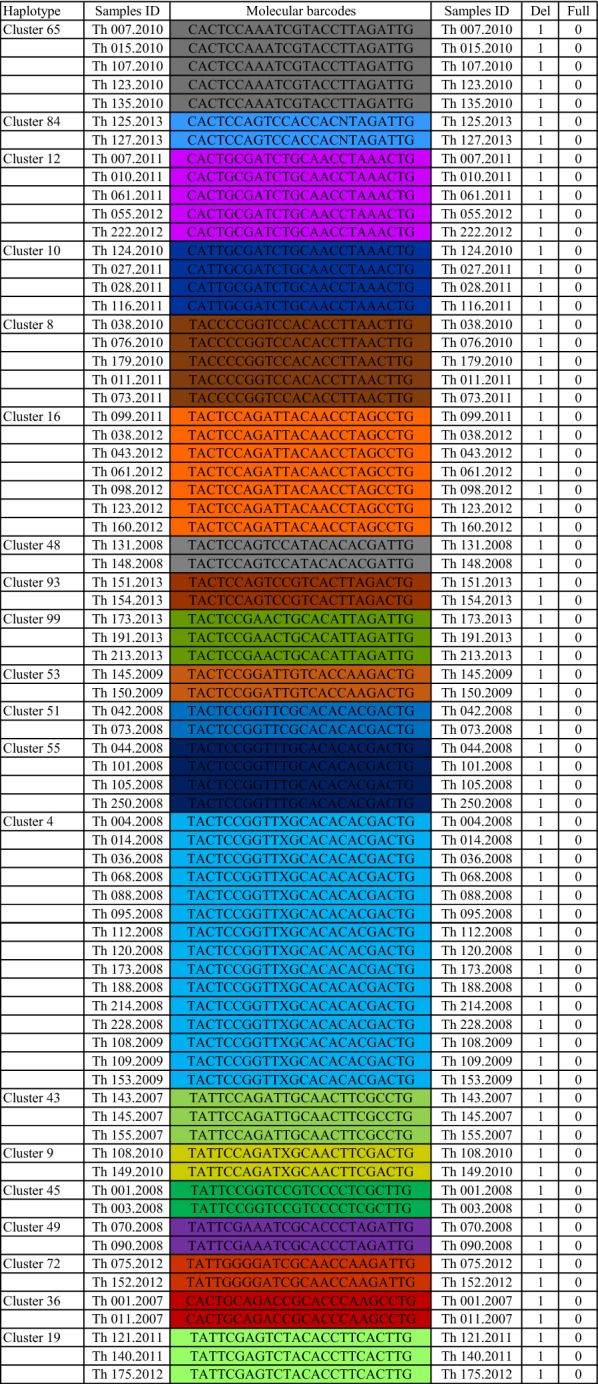
Frequency of the deletion over time within each cluster that contain only the deletion

## Discussion

*Plasmodium falciparum* reticulocyte binding protein homolog 2b (*PfRh2b*), has been reported to present a large structural polymorphism (0.58 kb deletion) in the c-terminal region. It is an important target of immunity [10] and could be considered as a potential candidate for blood stage vaccine development. Previous works showed that *PfRh2b* deletion was highly prevalent in isolates from Senegal and Africa [10, 26, 27] and present in different populations of the world [10]. However, malaria prevalence has been declining and interventions are taking place across sub-Saharan Africa. As these may be selecting for parasite subtypes, the changes imposed on this PfRh2b protein remains unknown. Therefore, the goal of this study was to follow the temporal evolution of the prevalence of *PfRh2b* deletion using samples from 2007 to 2013 in Thiès and from 1984 to 2013 in Western Gambia.

For the two populations, the prevalence of the deletion variant of *PfRh2b* ranged from 30 to 80%. However, while the prevalence of this deletion declined in Senegal, there was a steady increase in the Gambian populations studied until 2012. The differences in the trends between the two countries and within each country can be the result in random shifts in parasite allele frequencies in different geographic region due to genetic drift in isolated populations. Other explanations could be the genetic background of the host, the environmental modification, immune pressure, drug pressure and or drug resistance [28, 34].

Furthermore, the prevalence of *PfRh2b* deletion in different age groups in Thiès showed that the deletion was significantly less present in children compared to older patients. In Western Gambia, the prevalence of *PfRh2b* deletion was more common in younger children compared to older children, but the difference is not significant. With those results observed in these two sites, the association between age and deletion is not clear. However, a previous study did not find a relation between age and deletion [10].

Moreover, the evaluation of temporal variation of *PfRh2b* polymorphism in Senegal and Gambia populations was performed by using *Fst* to estimate the degree by which this locus is subject to a selection. Indeed, selection intensity can lead to differences in diversity and generate divergence among natural populations.

Thus, the *Fst* value (0.09) in Thiès, from 2007 to 2013 is higher than those observed for *PfRh2b, Msp2, EBA 175* and *Pfs48/45* within Senegal and between African countries [10]. However, this *Fst* value was lower than those observed for *Msp2* and *EBA 175* dimorphisms, which has been reported to exhibit less genetic differentiation and possible balancing selection in the global populations (African, South East Asian and Latin American populations) [10]. In Western Gambia, the *Fst* value was low and similar to that observed for the *EBA 175* dimorphism within Senegal, which has been shown to have also minimal genetic differentiation globally [10]. Overall, the *Fst* values obtained in this study indicate less genetic differentiation suggesting that *PfRh2b* polymorphism is under balancing selection over time in Thiès and Western Gambia.

Additionally, the prevalence of *PfRh2b* deletion according to the molecular barcode of isolates from Thiès was analysed to determine whether the deletion is associated with a specific barcode in this region. Analysis of the distribution of *PfRh2b* polymorphism in shared barcode clusters suggests that deletion is associated at some haplotype clusters in the population. However, the removal of large clusters from the population do not effect the prevalence of the deletion over time. The results of the study provide information on the genetic diversity of the *PfRh2b* gene that could be useful in the validation of this antigen as a potential vaccine candidate.

## Conclusion

Temporal trends in the frequency of the deleted *PfRh2b* variant differed in Senegal and The Gambia. This may suggest an effect of local factors on the prevalence of *PfRh2b* deletion between the years. Changes in the frequency of PfRh2b polymorphism over the time could be an obstacle to the implementation of this protein as a potential vaccine candidate as allele specific immunity may affect its efficacy. It will be important to investigate the natural antibodies responses against PfRh2b over the time in different malaria endemic countries to evaluate this antigen as a vaccine candidate.

## Additional file

**Additional file 1: Table S1.** Number of samples by year of *PfRh2b* polymorphism in Thiès and Western Gambia. Column N shows the number of samples analysed. n = the number of samples of each allele. *PfRh2bdel *= deletion present; *PfRh2bfull *= full-length sequence; Mix =* PfRh2bDel/PfRh2bfull*.

## Data Availability

The datasets generated and analysed during the current study are included within the article.

## Abbreviations

*PfRh2b*: *Plasmodium falciparum* reticulocyte binding protein homolog 2b
*PfRh2bdel*: *PfRh2b* deletion
*PfRh2bfull*: *PfRh2b full*-*length*
DBL: Duffy binding-like family
SNPs: single nucleotide polymorphisms
SLAP: Service de Lutte Anti-parasitaire
MRC: Medical Research Council
DNA: deoxyribonucleic acid
PCR: polymerase chain reaction
