# Supplementary material for: Temporal changes in Plasmodium falciparum reticulocyte binding protein homolog 2b (PfRh2b) in Senegal and The Gambia
Source: Malar J. 2019 Jul 16;18:239. doi: 10.1186/s12936-019-2868-x (PMC6636118; doi:10.1186/s12936-019-2868-x)
Supplement: Supplementary file 1 — Additional file 1: Table S1. Number of samples by year of PfRh2b polymorphism in Thiès and Western Gambia. Column N shows the number of samples analysed. n = the number of samples of each allele. PfRh2bdel = deletion present; PfRh2bfull = full-length sequence; Mix = PfRh2bDel/PfRh2bfull. [file 12936_2019_2868_MOESM1_ESM.docx]

| Sites | Years | N | Alleles | | |
| --- | --- | --- | --- | --- | --- |
|  |  |  | *PfRh2bDel* (n) | *PfRh2bfull* (n) | Mix (n) |
| THIES  Total | 2007  2008  2009  2010  2011  2012  2013 | 127  118  102  114  121  141  126  **849** | 78  75  58  41  43  57  43 | 36  39  41  67  74  75  73 | 13  4  3  6  4  9  10 |
| BRIKAMA  Total | 1984  2005  2007  2008  2010  2012  2013 | 56  75  99  120  45  53  63  **531** | 26  49  44  77  15  42  33 | 17  20  49  38  15  7  23 | 13  6  6  5  5  4  7 |
